# RPAP3: Structural evolution, chaperone networks, and disease implications of a transcriptional Co-chaperone

**DOI:** 10.1016/j.jbc.2026.111439

**Published:** 2026-04-09

**Authors:** Larissa M. Antonio, Carlos H.I. Ramos

**Affiliations:** 1 Institute of Chemistry, University of Campinas (UNICAMP), Campinas, São Paulo, Brazil; 2 National Institute of Science and Technology for Bioimage and Structural Biology INBEB, Rio de Janeiro, Rio de Janeiro, Brazil

**Keywords:** RPAP3, R2TP, protein homeostasis, Hsp90, PIH1D1

## Abstract

The functional versatility of Hsp90 relies on its association with specialized co-chaperones that regulate client recruitment and maturation. Among these, the R2TP complex, comprising RUVBL1, RUVBL2, RPAP3 (Tah1 in yeast), and PIH1D1, acts as a conserved assembly factor essential for the biogenesis of large multiprotein machineries, including RNA polymerases, snoRNPs, PIKKs, and mTOR signaling complexes. RPAP3 functions as a central scaffold within the R2TP-Hsp90 system, linking Hsp90 and Hsp70 to the RUVBL1/2 ATPase core through its TPR domains and C-terminal interaction with PIH1D1. This modular organization enables RPAP3 to integrate chaperone-mediated folding with client delivery and complex assembly. Notably, dysregulation of RPAP3 has been implicated in oncogenic processes, highlighting its biomedical relevance. This review synthesizes current structural, functional, and evolutionary insights into RPAP3, focusing on its role within the R2TP-Hsp90 machinery and its emerging connections to human disease.

The maintenance of protein homeostasis is crucial for cellular viability and relies on a dynamic network of chaperones and co-chaperones that ensure correct folding and assembly of macromolecular complexes (1, 2). Among these systems, the Hsp90-centered machinery plays a key role in the maturation and stabilization of numerous client proteins involved in transcription, signal transduction, and genome maintenance (3). Because protein-protein interactions (PPIs) underpin the organization of cellular pathways, elucidating how Hsp90 recruits and assembles client complexes has become central to understanding proteostasis (4, 5).

Hsp90 is a dynamic homodimer composed of three highly conserved domains: an N-terminal domain (NTD), which binds nucleotide; a middle domain (MD), which forms the primary client binding site; and a C-terminal domain (CTD), which mediates dimerization and stabilizes the characteristic V-shaped conformation of the chaperone (6). The extreme C-terminus additionally contains a conserved MEEVD motif that is specifically recognized by tetratricopeptide repeat (TPR)-containing co-chaperones. Hsp90 activity is ATP-dependent and further modulated by numerous co-chaperones, including Tom70 (translocase of the mitochondrial outer membrane), CHIP (carboxyl terminus of Hsp70 interacting protein), SGT (small glutamine-rich TPR-containing protein), HOP (Hsp70-Hsp90 organizing protein), and PP5 (protein phosphatase 5), which promote the folding or degradation of a distinct subset of client proteins (7, 8, 9, 10). Consequently, Hsp90 functions as a central hub in the cellular proteostasis network, facilitating the folding and maturation of numerous client proteins and associating with approximately 10% of the eukaryotic proteome (11).

Within the Hsp90 network, the R2TP complex, composed of RUVBL1, RUVBL2, RPAP3/Tah1, and PIH1D1 (12) (Fig. 1), is a conserved co-chaperone assembly factor in higher eukaryotes that promotes the formation of multiprotein machineries. These include RNA polymerases (13, 14), small nucleolar ribonucleoproteins (snoRNPs) (15, 16), PIKKs (phosphatidylinositol-3-kinase-like kinases) (17, 18, 19, 20, 21), and the mTOR/TSC signaling complex (14, 22). Together with additional components such as WDR92, RPB5, and prefoldin, R2TP forms a broader chaperone-like machine assembly known as the PAQosome (particle for arrangement of quaternary structure), which coordinates the biogenesis and stabilization of diverse macromolecular complexes (12).

**Figure 1:**
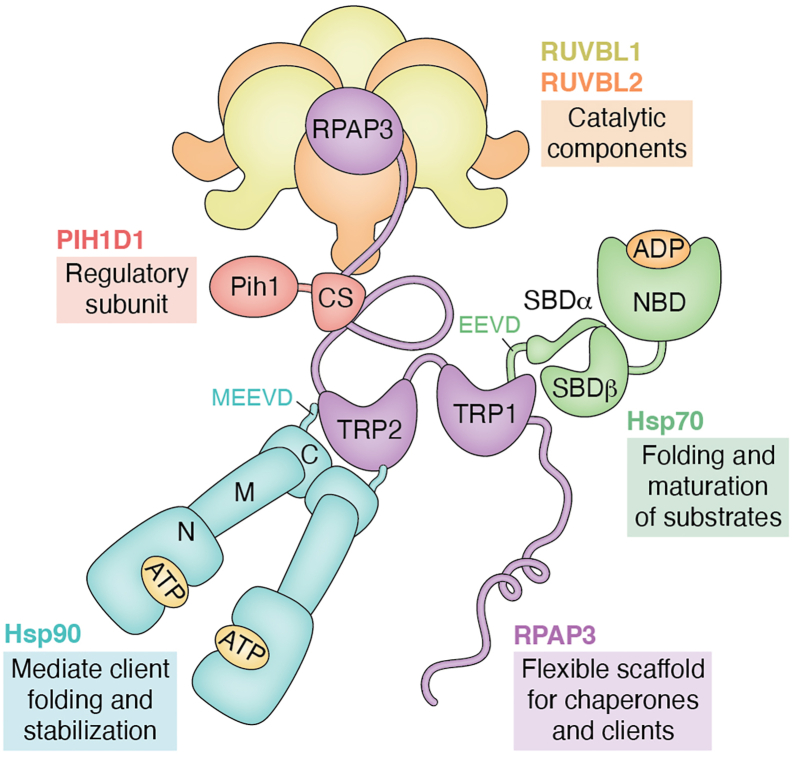
**Position of RPAP3 within the R2TP–Hsp90 chaperone network.** Within this cooperative chaperone system, RPAP3 engages both Hsp70 (*green*; nucleotide binding domain, NBD, and substrate binding domain, SBD, are shown) and Hsp90 (*blue*; N-terminal, Middle, and C-terminal domains are shown) that contain a C-terminal EEVD motif, through its TPR1 and TPR2 domains. In parallel, RPAP3 connects to the RUVBL1/2 ATPase complex *via* its C-terminal interaction with PIH1D1 (*red*; Pih1 and CS domains are shown). Together, these interactions support a model in which the R2TP complex receives client proteins from Hsp70/Hsp90 and promotes their assembly into higher-order macromolecular complexes (46, 49). The primary function of each protein is highlighted in its corresponding box.

Large-scale proteomic and genomic screens in *Saccharomyces cerevisiae* identified Tah1 (TPR-containing protein associated with Hsp90) (3, 23). In higher eukaryotes, its ortholog RPAP3 (RNA polymerase II-associated protein 3) acts as a multi-linker scaffold that links Hsp90 to the R2TP complex. The TPR domain of RPAP3 binds the MEEVD motif of Hsp90, whereas its C-terminal and PIH1D1-binding regions coordinate client recognition and recruitment. This modular architecture enables RPAP3 to modulate R2TP activity and operate at the interface between protein folding and transcriptional regulation.

Although mainly characterized in human cells, recent structural and comparative studies indicate that the architecture and function of RPAP3 vary across eukaryotes (24). Differences in domain composition and partner specificity among orthologs emphasize the evolutionary plasticity of this protein. Moreover, RPAP3 dysfunction has been associated with oncogenic contexts, highlighting its biomedical relevance (8, 16).

This review provides a comprehensive overview of current knowledge on RPAP3, emphasizing its structural features, evolutionary conservation, interaction networks within the R2TP-Hsp90 system, and emerging connections to disease associations.

## Structural features and domain organization

### TPR domain

The tetratricopeptide repeat (TPR) domain typically comprises 3 to 16 tandem repeats of a 34-residue consensus sequence, with each motif adopting a canonical helix-turn-helix conformation. This evolutionarily conserved motif is found in all kingdoms of life and plays essential roles in mediating protein–protein interactions (25, 26).

**Figure 2:**
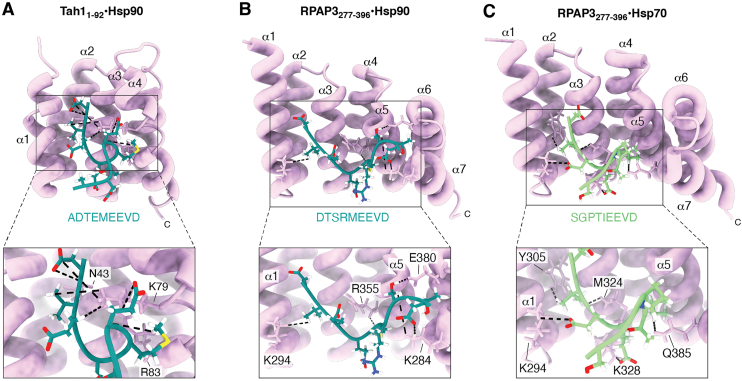
**Structural representation of TPR domain interactions with Hsp90 and Hsp70.***A*, TPR domain of Tah1 (residues 1–92; lilac) in complex with the C-terminal Hsp90 peptide ADTEMEEVD^709^ (blue sticks; PDB: 2LSV). *B and C*, TPR2 domain of RPAP3 (residues 277–396; lilac) bound to the C-terminal peptides of human Hsp90α DTSRMEEVD^732^ ((*B*) *blue sticks*; PDB ID: 6FDP) and Hsp70 SGPTIEEVD^646^ ((*C*) *green sticks*; PDB ID: 6FDT). Key RPAP3 residues in RPAP3 are shown in *stick* representation, derived from the lowest-energy NMR structure. Inset: Close-up view of key TPR residues (shown as *lilac sticks*) interacting with the chaperone motif through a carboxylate clamp.

Numerous crystallographic structures of TPR-containing proteins deposited in the Protein Data Bank (PDB) have revealed that each TPR motif forms an antiparallel α-helical hairpin. The tandem arrangement of these hairpins generates an amphipathic groove that serves as a versatile scaffold for intra- and intermolecular interactions (27). High-resolution structural analyses have shown that TPR domains specifically recognize the C-terminal MEEVD pentapeptide of Hsp90 (Fig. 2), a canonical docking motif shared by many TPR co-chaperones (8, 28, 29).

The groove of the TPR domain is lined with positively charged residues that interact electrostatically with the acidic Glu-Asp pair of the Hsp90 MEEVD motif, while hydrophobic contacts further stabilize the complex. Together, these interactions form a distinctive “carboxylate clamp” mediated by conserved residues within the TPR domain (28, 30).

RPAP3 functions as a central scaffold within the R2TP co-chaperone complex, providing multiple protein-interaction interfaces, mainly through its TPR domains, that coordinate client recruitment and assembly. To introduce the features and implications of its TPR domain structure more clearly, it is useful first to examine a simpler, well-characterized ortholog. Structurally, Tah1 from *S. cerevisiae* is a small monomeric protein composed of 111 amino acids and contains a single TPR domain followed by an unstructured C-terminal region (24).

The NMR structure of Tah1 (PDB 2LSU) shows that it contains a structured TPR domain (residues 1–92) composed of two TPR motifs (TPR1 and TPR2), followed by a flexible C-terminal region (residues 93–111) (29, 31). TPR1 comprises helices 1A and 1B and contains several residues that are conserved among canonical TPR motifs, whereas TPR2 comprises helices 2A and 2B and is more degenerate. A third helix, helix C, connects the TPR2 to the flexible C-terminal region and does not possess the defining features of a TPR helix. Nevertheless, helix C appears to stabilize the TPR domain in free Tah1.

The yeast Hsp90 ADTEMEEVD^709^ peptide specifically interacts with Tah1 TPR domain (32). This complex has been solved by NMR (PDB 2LSV) and is shown in Figure 2*A*. In this structure, Tah1 closely resembles its unbound conformation, except for helix C, which moves slightly towards helix 2A (29). Indeed, helix C appears to be critical for binding: its deletion abolishes the interaction, and mutations in key residues of helix C (Lys79, Tyr82, and Arg83) eliminate Hsp90 binding. The effects of these substitutions on Hsp90 peptide affinity have been measured *in vitro* using isothermal titration calorimetry (ITC) (32, 33). The impact of mutations in the Hsp90 peptide was also investigated, showing that substitution of D709 completely disrupts binding, whereas substitution at E706 or V708 substantially reduces the interaction. Notably, the carboxylate clamp residues in Tah1 (Lys8, Asn12, Asn43, Lys79, and Arg83) are equivalent to those found in the TPR domains of other co-chaperones, such as CHIP and HOP (TPR2A-bound structures) (29).

Upon binding to Hsp90, Tah1 has been reported to dimerize, thereby preventing nonspecific interactions with other TPR co-chaperones. Because Tah1 contains only one TPR domain while Hsp90 is a dimer bearing two MEEVD motifs, the resulting complex adopts a 1:1 stoichiometry (Tah1 dimer: Hsp90 dimer) (34). However, other biophysical and structural studies have reported that Tah1 displays only weak dimerization in solution, and no stable dimer has been observed in crystal or NMR structures (32, 35).

Significant divergence is observed among Tah1 orthologs. In higher eukaryotes, the RPAP3 subunit is a much larger protein organized into two tandem TPR domains (TPR1 and TPR2), followed by an unstructured region and an additional C-terminal domain, namely RPAP3 (36, 37). The presence of two consecutive TPR domains, rather than a single TPR as seen in yeast Tah1, likely enhances RPAP3’s ability to coordinate multiple simultaneous interactions, reflecting an evolutionary adaptation toward increased network complexity.

Notably, TPR domains can bind not only to Hsp90 but also to Hsp70. This dual specificity is observed in both RPAP3 (37, 38) and its yeast homolog Tah1 (39). In human RPAP3, the two TPR domains are designated TPR1 (residues 133–255) and TPR2 (residues 281–396). Both domains comprise seven alpha-helices and are capable of binding the EEVD residues of Hsp70/Hsp90 (40) (Fig. 2, *B* and *C*). NMR studies have highlighted the importance of residues K286, N290, N321, M324, L327, K328, G347, K351, R355, T358, and Q385 within TPR2 for Hsp90 binding (37).

Consistent with structural observations (37), RPAP3-TPR2 binds Hsp90 with substantially higher affinity than Hsp70; the affinity for Hsp70 is approximately 10-fold lower than for Hsp90. In the same NMR study, residues K294 and Q385 of TPR2 were identified as key determinants of Hsp70 binding (37). Conversely, no strong interactions were detected between TPR1 and either Hsp70 or Hsp90, and the authors suggested that differences at non-conserved positions, such as K384 in TPR2, may underlie this reduced affinity. Overall, these data indicate that TPR2 exhibits a particularly high affinity for Hsp90, approximately 20-fold higher than that of TPR1. In contrast, Pal *et al.* (2014) (20) reported that TPR1 binds Hsp90 with greater affinity than TPR2. Thus, additional studies are required to determine whether there is an inherent binding preference between the two RPAP3 TPR domains or if cellular context, post-translational modifications, or client load modulate binding properties.

Additionally, mass spectrometry and ITC analyses indicate that a dimeric Hsp90 can accommodate up to two RPAP3 molecules (37). Nonetheless, beyond its role in Hsp90 binding, biochemical evidence suggests that the TPR domains of RPAP3 may also contribute to the recruitment of additional co-chaperones or client proteins. These domains are therefore likely to act as initial docking elements that localize RPAP3 to active Hsp70/Hsp90 complexes and position R2TP for effective client transfer and assembly.

### Intrinsically disordered region

The TPR modules and the C-terminal domain are connected by an intrinsically disordered region (IDR) region. This conformationally flexible segment is thought to play a regulatory role, acting as a mechanical hinge that enables RPAP3 to adjust the spatial arrangement of residues 430 to 441 and 492 to 500 within the IDR of the human RPAP3 isoform 1 during the contacts with RUVBL1 (41).

Bioinformatic analyses, including AlphaFold3 structural predictions and disorder-propensity profiling, indicate that the RPAP3 linker is enriched in disorder-promoting residues, such as glycine, serine, and proline. This amino acid composition confers high intrinsic flexibility. These disordered segments likely function as molecular “entropic springs,” allowing the TPR domains to sample multiple relative orientations and thereby optimize binding efficiency (42).

**Figure 3:**
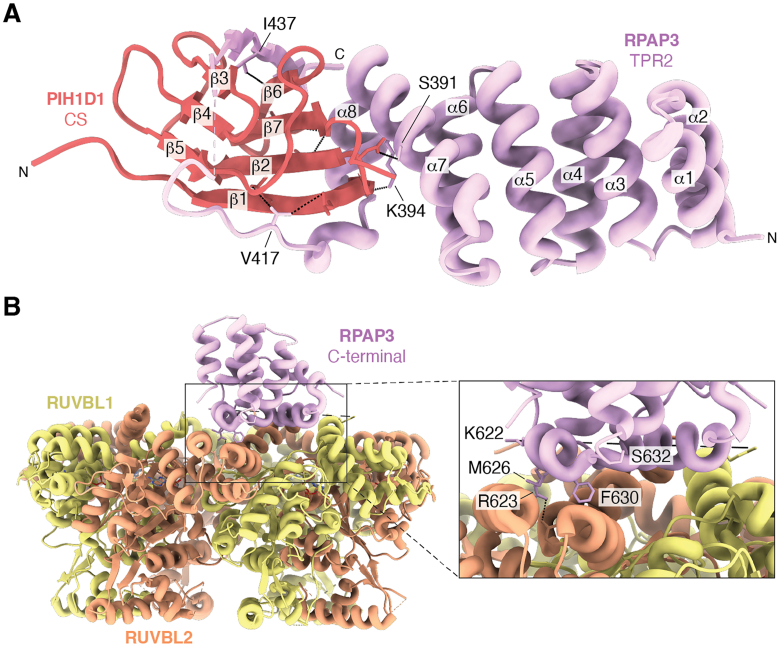
**Structural representation of the major RPAP3 interaction interfaces.***A*, cartoon representation of the complex between the CS domain of PIH1D1 (residues 199–290; *red*) and the TPR2–isoform region of RPAP3 (residues 281–445; *lilac*) (PDB ID: 6GXZ). *B*, Cryo-EM structure of the RUVBL1–RUVBL2–RPAP3 complex. A close-up view highlights the contact interface between the RPAP3 C-terminal domain and RUVBL2, emphasizing key interacting residues (PDB ID: 6FO1).

RPAP3 isoform 1 contains an additional 34 residues compared to Isoform 2, introduced by alternative splicing. These residues (397–429) are located between TPR2 and the IDR and are important for PIH1D1 binding (43). Human PIH1D1 spans two principal domains: (i) a conserved PIH (phosphopeptide-interacting) domain that specifically recognizes phosphorylated substrates and contains a conserved DpSDD motif, and (ii) a CS domain that mediates binding to RPAP3 (18, 20, 44). Crystallographic and Nuclear Overhauser effects (NOE) NMR conformational ensemble of RPAP3 residues 281 to 445 in complex with PIH1D1 residues 199 to 290 have been determined (37), revealing a contribution of disordered regions to the interaction surface (Fig. 3*A*). Seraphim *et al.* (2022) (41) further demonstrated that residues 416 to 429 within the RPAP3 isoform 1 IDR mediate binding to the PIH1D1 CS domain and form intermolecular β strands. This finding partially aligns with previous data indicating that the loop surrounding the CS domain is not necessary for the heterodimer formation (37).

### C-terminal region (CD)

The C-terminal domain of RPAP3 (residues 540–665), also referred to as CD (36, 37, 45), is absent in Tah1. This domain is composed of eight α-helices, numbered 1 to 8, which pack together to form a compact, globular fold. Its interaction with RUVBL2 is crucial for R2TP assembly (Fig. 3). Helices 1 to 6 of the RPAP3 C-terminal domain (CD-RPAP3), as well as several residues in the hydrophobic core, are the most conserved. Helix 6 interacts primarily with RUVBL2, whereas helix 1 contacts both RUVBL1 and RUVBL2; consistently, mutations in helix 6 abolish the interaction of RPAP3 with RUVBL1/2 (45). The C-terminal domain also contributes to binding MONAD. Comparison of the RPAP3 structure bound within the R2TP complex with that of free RPAP3 in solution reveals only minor differences, mainly in the side-chain conformations of a subset of residues located in the core of this α-helical domain.

Cryo-electron microscopy (cryo-EM), combined with cross-linking mass spectrometry (XL-MS), of the human R2TP complex unexpectedly revealed that the ATPase face of each RUVBL2 subunit interacts with the RPAP3 C-terminal domain (Fig. 3*B*) (41, 46). The cryo-EM structure further demonstrated that a single RUVBL1/2 hexamer can accommodate up to three RPAP3 molecules, one bound per RUVBL2 subunit. Moreover, cryo-EM studies have shown that the PIH1D1-RPAP3 heterodimer is positioned on top of the RUVBL1/2 hexameric ring and is connected through flexible linkers that permit rotational freedom (36, 46). This mobility likely enables the R2TP complex to accommodate substrates of varying sizes and architectures, such as the large RNA polymerase II complex *versus* smaller nucleolar RNPs.

Additionally, WD repeat domain 92 (WDR92) interacts with RPAP3 *via* its C-terminal domain and mediates R2TP association with the Prefoldin module (47, 48). The C-terminal region of RPAP3 might also engage additional adaptor proteins involved in PIKK complex assembly, although direct structural evidence for these interactions remains limited (21, 49).

Proteomic analyses of an RPAP3 C-terminal fragment (residues 535–665) expressed in HeLa cells identified multiple interaction partners, including SHQ1 and NOP58 (components of snoRNPs), PRPF8 and U5 snRNP components (splicing machinery), POLR2A (a subunit of RNA polymerase II), ZNHIT2 (an R2TP cofactor), and PFDN2 (a PAQosome subunit). Moreover, cooperative interactions between RUVBL1/2 and the RPAP3 C-terminal domain promote the association of additional clients NOPCHAP1, WDCP, and DPCD (45). These findings highlight the RPAP3 C-terminal as a versatile scaffold that facilitates the maturation of diverse macromolecular assemblies.

Together, the modular architecture of these interactions enables R2TP to integrate multiple proteostatic pathways simultaneously, coupling Hsp90’s folding capacity with RUVBL1/2 remodeling events and providing a dynamic assembly platform for quaternary structure assembly. Consequently, obtaining a high-resolution structure of the full-length RPAP3 is challenging. RPAP3 behaves as a dynamic, elongated protein in solution and has an extensive intrinsically disordered region, as revealed by small-angle X-ray scattering (SAXS) analysis (41).

## Evolutionary conservation of RPAP3 orthologs

**Figure 4:**
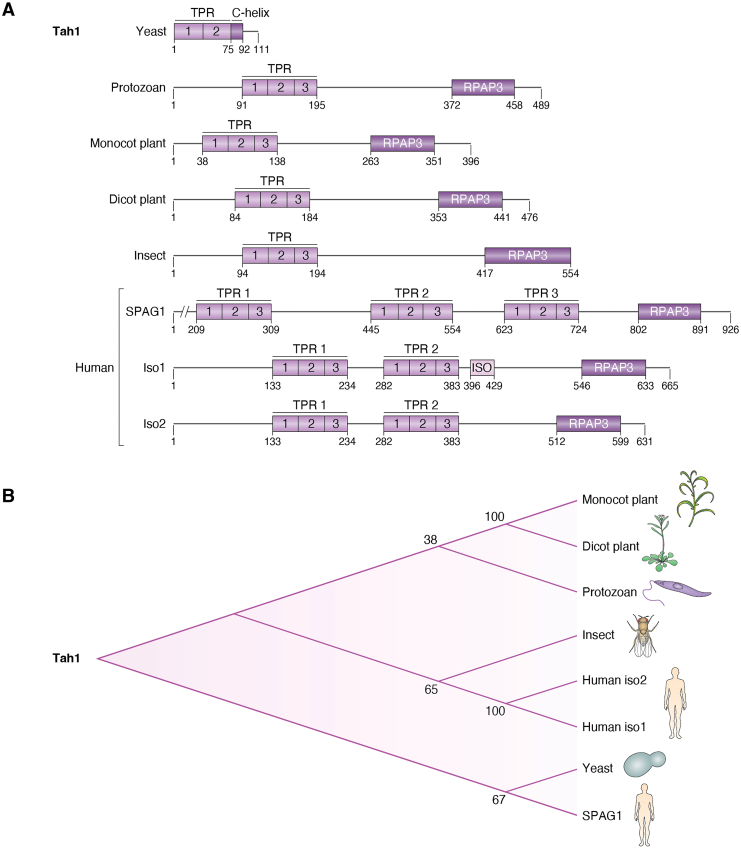
**Comparative analysis of RPAP3 orthologs.***A*, Structural domains and architecture of Tah1 and representative orthologs across eukaryotes. *B*, phylogenetic tree. Phylogenetic tree constructed using MEGA 12 based on representative sequences from yeast (*Saccharomyces cerevisiae*, NCBI: NP_009986.1), protozoan (*Leishmania major*, NCBI: XP_003722051.1), monocot plant (*Sorghum bicolor*, NCBI: XP_002440525.1), dicot plant (*Arabidopsis thaliana*, NCBI: CAA0299393.1), insect (*Drosophila melanogaster*, NCBI: NP_524664.1), and human (*Homo sapiens* isoform 1, NCBI: NP_078880.2; isoform 2, NCBI: NP_001139547.1; and SPAG1, NCBI: NP_001361250.1). Numbers at the nodes represent bootstrap values obtained from 1000 replicates, indicating the statistical support for each branch.

RPAP3 orthologs are widely distributed across eukaryotes, and comparative analyses (Fig. 4) indicate that, despite differences in overall size, the proteins exhibit high homology and a conserved domain architecture. For instance, the core TPR domains share about 40% identity between yeast Tah1 and human RPAP3, reflecting strong evolutionary pressure to maintain the carboxylate clamp and the EEVD-binding motifs, which are essential for Hsp90 interaction. This sequence conservation also includes the PIH1D1-binding regions, where key residues involved in β-strand formation and CS domain recognition are preserved across metazoans, underscoring the functional importance of R2TP complex stability (37, 41).

Partner specificity also evolves; for instance, while Tah1 primarily interfaces with Pih1 (yeast PIH1D1 ortholog) (3), mammalian RPAP3 engages additional adaptors like WDR92 and prefoldin components, facilitating integration into the expanded PAQosome (29, 47, 48). These modifications, which gain in modular domains and shifts in interaction networks, suggest adaptive divergence to support increasing cellular complexity from unicellular to multicellular organisms. Such evolutionary patterns highlight RPAP3's role as a dynamic scaffold, where conserved sequence elements ensure core chaperone coupling, while domain gains and partner expansions enable specialized functions in diverse proteomes.

### Yeast (*S. cerevisiae*): The minimalist scaffold Tah1

The yeast ortholog Tah1 (TPR-containing protein associated with Hsp90) is the simplest RPAP3 counterpart. Its domain structure comprises a single N-terminal TPR domain (residues 1–75) followed by a short C-terminal tail (Fig. 4) (3, 32). Tah1 binds Hsp90 through its canonical TPR groove, whereas a C-terminal α-helix is sufficient for binding Pih1, the yeast PIH1D1 ortholog (20). Because Tah1 lacks the C-terminal RPAP3 domain, its association with RUVBL1/2 is mediated through Pih1 (50). In addition, Tah1 lacks the extended IDR found in higher orthologs, resulting in a more compact architecture, consistent with a simpler proteostatic network in unicellular fungi (24).

Functionally, Tah1 primarily assists in the assembly of snoRNPs, which are essential for ribosome biogenesis, and RNA polymerase II. However, it seems to lack the versatility required to handle larger or more intricate clients, such as PIKKs (18). In contrast to RPAP3, the absence of a central flexible region and additional interaction nodules restricts conformational adaptability, making Tah1 more like a relatively rigid “bolt” than a dynamic scaffold. From an evolutionary perspective, the simplicity of Tah1 likely reflects the reduced chaperone demands of unicellular fungi, which possess fewer and less diverse client repertoires. Consistent with this idea, deletion of Tah1 in *S. cerevisiae* is non-lethal under standard growth conditions but becomes detrimental under heat or nutrient stress, indicating an auxiliary role in stress adaptation rather than an essential housekeeping function (31).

### Protozoans

Searches for Tah1 orthologs in protozoans, such as *Plasmodium* and *Leishmania*, have identified genes encoding TPR domains similar to those in Tah1 (51, 52). In *Leishmania donovani*, the putative ortholog shows greater overall similarity to human RPAP3 than to yeast Tah1. A comparable pattern is observed in *Plasmodium falciparum*, where two genes encode proteins containing two distinct TPR domains, reminiscent of the tandem TPR organization characteristic of RPAP3.

In *Leishmania major*, the gene designated LmTPR is expressed across all developmental stages (53). LmTPR harbors a single predicted TPR domain spanning residues 91 to 195, and the purified recombinant protein, a 489-residue monomer, consistently interacts with both Hsp90 and Hsp70 *in vitro*. Sequence analyses further indicate that LmTPR contains a C-terminal RPAP3-like domain (residues 372–458). However, additional biochemical and structural studies are required to determine whether this region can bind RUVBL1/2 and PIH1D1 and thus functionally recapitulate the human RPAP3 C-terminal module.

Blast analyses (53) showed that homologues of LmTPR are present in all *Leishmania* species examined (*L. donovani*, *Leishmania infantum*, *Leishmania mexicana*, *Leishmania tarentolae*, *Leishmania guyanensis*, *Leishmania panamensis*, and *Leishmania braziliensis*) and are highly conserved at the sequence level. Altogether, these observations indicate that LmTPR is conserved within the *Leishmania* genus and support the hypothesis that this protein plays an important role in connecting the R2TP/Hsp90 system with specific protozoan clients.

### Insects (*Drosophila melanogaster*): intermediate complexity

In *D. melanogaster*, Spaghetti (Spag; gene CG13570, UniProt Q9V3E9) encodes a protein similar to mammalian RPAP3 (38) and exhibits an intermediate level of complexity between yeast Tah1 and vertebrate RPAP3 orthologs. Most insect Spag proteins have domain expansion, containing a single N-terminal TPR domain followed by a central region predicted to be intrinsically disordered and a putative RPAP3-like domain at the C-terminus. In *Drosophila*, Spag is 534 residues long, with the TPR domain located approximately between residues 94 to 124, followed by a predicted disordered region, and a C-terminal RPAP3-like domain extending from residues 417 to 554 (Fig. 4) (38).

Co-immunoprecipitation experiments in the *Drosophila* S2 cells using 3xFLAG-tagged PIH1D1 demonstrated that endogenous Spag is part of a multimeric Hsp90 co-chaperone R2TP complex. Consistent with an essential role, *spaghetti* mutations are lethal: maternal contribution allows null mutant embryos to initiate development, but larvae die with widespread organ atrophy, reflecting Spag’s multifunctional co-chaperone functions. Functionally, Spag is required for ovarian germline stem cell (GSC) maintenance (54), regulation of circadian rhythm (55), basement membrane formation (56), and during fly development, as well as assembly of cellular machineries (38).

In summary, protozoan orthologs like LmTPR and insect orthologs like Spag represent evolutionary intermediates that are more complex and essential than the minimalist yeast Tah1, yet less structurally elaborate than mammalian RPAP3, while already integrating R2TP-Hsp90 functions into multiple developmental and physiological pathways. These features suggest progressive domain acquisition to support increasingly complex chaperone networks.

### Plants: formation of R2T

In plants, RPAP3 orthologs typically retain a domain organization similar to that of metazoan RPAP3. RPAP3 in *Arabidopsis thaliana* (At) (AGI code AT1G56440, UNIPROT Q5XF05) is 476 residues long and comprises a TPR domain (residues 84–184), a predicted intrinsically disordered region (residues 252–322), and an RPAP3-like C-terminal domain (residues 353–441). This protein was identified in the purified R2T/prefoldin complex from *Arabidopsis* cell suspensions (57). RPAP3 localizes to both the nucleus and the cytoplasm of leaf cells from *Arabidopsis* (58) and *Nicotiana benthamiana* (57).

The TPR domains of RPAP3 from *A. thaliana* (AtRPAP3) and *Sorghum bicolor* (SbRPAP3) show significant sequence homology to the TPR2 domain of human RPAP3, which mediates Hsp90 interaction. Accordingly, the role of RPAP3 in recruiting Hsp90 and acting as an allosteric regulator appears to be conserved in plants. SbRPAP3 interacts with human RUVBL1/2 in heterologous assays and binds Hsp70, albeit with lower affinity, forming a 1:1 monomeric complex (57, 59). SbRPAP3 shares 50%, 74%, and 93% identity with its orthologs from *A. thaliana*, *Triticum aestivum*, and *Zea mays*, respectively. SbRPAP3 has 396 residues and is an elongated monomer in solution (59).

Cryo-EM analyses of the *Arabidopsis* complex reveal a distinct architecture comprising a dodecameric assembly of AtRUVBL1 and AtRUVBL2, with a single AtRPAP3 molecule bound to the AAA+ face of one AtRUVBL2 subunit, exhibiting relatively low affinity. Like its orthologs, AtRPAP3 interacts with RUVBL1/2-Hsp90 client complexes, suggesting that R2T facilitates client transfer and may modulate chaperone activity. Despite these interactions, AtRPAP3 does not stimulate the ATPase activity of the AtRUVBL1/2 complex, a finding consistent with observations in the human ortholog (41, 57).

Within the green lineage, homologs of Pih1/PIH1D1 appear to be restricted only to bryophytes and are absent from seed plants and other species without motile cilia (45, 57). In *Marchantia polymorpha*, Pih1/PIH1D1 expression is confined to the male reproductive organ (60). The apparent absence of a canonical R2TP complex in seed plants, together with the restricted expression of Pih1/PIH1D1 in the male sex organ of non-seed plants, implies that the R2T complex has largely taken over the cellular roles of R2TP in most plant lineages (57). Supporting this functional relevance, AtRPAP3 participates in developmental processes, especially in directional cell division within root meristems (58, 61).

### Mammals: Complex orchestration of client assembly

Mammalian RPAP3 represents the most elaborate form of this protein family, integrating multiple structural and regulatory innovations to coordinate the folding and assembly of diverse macromolecular complexes. Human RPAP3 exemplifies this complexity. Its domain organization comprises two N-terminal TPR domains followed by an elongated intrinsically disordered region and a structured C-terminal RPAP3 domain (Fig. 4) (62). Three human RPAP3 isoforms have been described. Isoform 1 (and possibly isoform 3) contains the whole segment required for engaging the CS domain of PIH1D1, whereas isoform 2 lacks 34 amino acids necessary for this binding, which may act as a negative regulator or competitor of the canonical R2TP function (45, 47).

Both TPR1 and TPR2 of RPAP3 interact with Hsp70 with moderate affinity, but RPAP3-TPR2 displays a markedly high affinity for Hsp90-derived peptides, approximately 20-fold higher than TPR1 in some assays. This enhanced affinity is attributed, at least in part, to the Met residue within the Hsp90 C-terminal binding motif that inserts more deeply into the TPR pocket, positioning it with respect to α helices 3 and 5, and stabilizing the complex, more effectively than the Ile residue in Hsp70, which is positioned with respect to α helices 5 and 7 (37). Such differential binding properties likely contribute to a hierarchical recruitment of Hsp70 and Hsp90 during client maturation.

In HEK293 cells, *RPAP3* overexpression enhances apoptosis and caspase-3 activation in response to tumor necrosis factor-α (TNF-α) plus cycloheximide (CHX) treatment (48). Conversely, CRISPR-Cas9-mediated knockout of *rpap3* in RPE-1 cells causes pronounced defects in ciliogenesis, reducing the proportion of ciliated cells, while exerting only minor effects on cilium length and without significantly altering the cell cycle profile (63).

Human RPAP3 accommodates a broad spectrum of clients by coordinating the assembly of PIKKs (mTOR, ATM, ATR), snoRNPs, and RNA polymerases I/II/III through the collaborative recruitment of adaptor proteins like TELO2 and WDR92 (18, 21). Regulatory mechanisms further modulate its activity, including phosphorylation sites (*e.g.*, Ser 626), and intrinsically disordered linker regions that enable allosteric control by kinases, such as CK2 and PLK1, thereby modulating interactions with RUVBL1/2, PIH1D1, and downstream clients (37, 41, 45). Additional phosphorylation sites in RPAP3 include serine at positions 116, 119, and 121, which are likely phosphorylated by CKS (64). Mutational analyses indicate that in the unphosphorylated state, RPAP3 shows increased association with pre-ribosomal structural proteins and proteins involved in ribosome maturation. In contrast, phosphomimetic mutants display enhanced association with ribosomal proteins and biogenesis factors linked to both the 40S and 60S ribosomal subunits. These layers of regulation integrate RPAP3 into broader signaling networks.

The expanded scaffold capacity of mammalian RPAP3 supports the high-fidelity assembly of large macromolecular complexes essential for development, immunity, and genome stability. From an evolutionary perspective, mammalian RPAP3 represents a culmination of structural complexity and functional versatility within this protein family, enabling the R2TP-Hsp90 system to support a broad range of essential cellular processes, from gene expression to genome maintenance.

### SPAG1: a metazoan RPAP3-like paralog

Sperm-associated antigen 1 (SPAG1 - UniProt Q07617) is involved in the biogenesis, structure, and regulation of cytoplasmic axonemal dynein arm assembly (65). Beyond its ciliary role, SPAG1 localizes to meiotic spindles, where its depletion severely impairs entry into the M-phase. Moreover, SPAG1 expression is upregulated in breast and lung cancers compared with normal tissues, suggesting a potential role in tumorigenesis (66).

SPAG1 shares a broadly similar modular organization with RPAP3. It contains nine TPR motifs arranged into three TPR domains and has an RPAP3-like C-terminal domain (65). Despite the similarities, SPAG1 is longer than RPAP3 due to its additional TPR domain. Amino acid sequence alignment indicates that the TPR1 domains are conserved between RPAP3 and SPAG1, whereas the TPR2 domain of RPAP3 is most similar to the TPR3 domain of SPAG1.

Evolutionary analyses suggest that SPAG1 and RPAP3 arose from a gene duplication event near the origin of Metazoa, accompanied by a parallel duplication that gave rise to PIH1D1 and PIH1D2 (45, 67). SPAG1 exhibits partial functional overlap with RPAP3, including binding to DNAAF2, a co-chaperone containing both PIH and CS domains, although with lower affinity than RPAP3 (45). Notably, the R2SP complex associates with Hsp70 chaperones and has been shown to interact with liprin-α2, a scaffolding protein involved in cell adhesion and migration. In this context, SPAG1 engages liprin-α2 partners, while PIH1D2 interacts directly with liprin-α2 and its downstream targets, exemplifying a modular client-handling mechanism adapted to metazoan-specific cellular functions (45).

### Evolutionary trajectory and functional implications

As discussed above, the evolution of RPAP3 and its paralogs reveals a trajectory of increasing structural complexity and functional diversity. In ancestral eukaryotes, RPAP3-like proteins likely functioned as minimal TPR adaptors, providing a basic scaffold for R2TP-mediated chaperone activities. This is exemplified by the compact TPR protein Tah1 in budding yeast. With the emergence of multicellularity, these proteins expanded both structurally and functionally, acquiring additional TPR domains, flexible interdomain linkers, and regulatory motifs that enabled the recruitment of a more diverse set of clients and integration into broader cellular networks.

This evolutionary expansion continued across metazoans, culminating in the sophisticated architecture of mammalian RPAP3. Parallel diversification events, such as the emergence of SPAG1 and lineage-specific adaptations in protozoans and plants, highlight how RPAP3 family proteins have evolved to meet the demands of increasingly complex proteostasis networks. Overall, RPAP3 and its orthologs demonstrate how modular domain architectures and intrinsically disordered linkers act as evolutionary substrates for functional innovation. Each structural elaboration enhanced the capacity for client recognition, signaling integration, and responsiveness to environmental and developmental cues.

## Interaction networks: R2TP-Hsp90 cooperativity and client proteins

### Coordination between Hsp90 and RUVBL1/2 through RPAP3-PIH1D1

**Figure 5:**
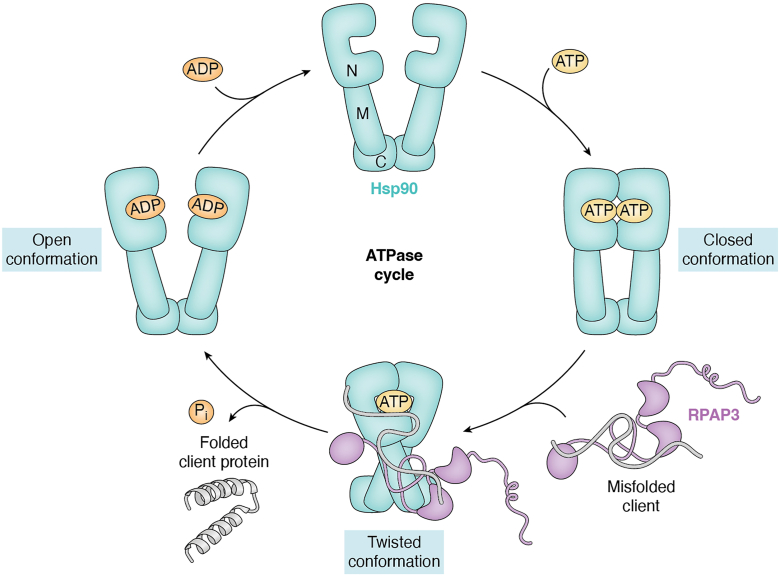
**Schematic representation of the Hsp90 chaperone cycle.** (1) Hsp90 recruits client proteins through the co-chaperone RPAP3 *via* interaction with the C-terminal MEEVD motif. (2) ATP binding induces a conformational transition to the closed state, trapping the client for folding or complex assembly. (3) Co-chaperones, such as RPAP3, stabilize this conformation and promote ATP hydrolysis through a transient “twisted” state, leading to client maturation and release. (4) ADP dissociation resets Hsp90 to the open conformation, enabling a new chaperoning cycle.

The R2TP complex functions as an Hsp90-dependent assembly platform, bringing together molecular chaperones, scaffolding adaptors, and remodeling ATPases to stabilize nascent macromolecular complexes. This coordination effectively couples the ATP-driven conformational cycle of Hsp90 with the remodeling activity of RUVBL1/2, creating a dynamic, dual-engine system capable of driving client folding, stabilization, and assembly (Fig. 5). In its simplest form, this cycle can be summarized as follows (20, 46, 49): (1) Hsp90 recognizes and binds a partially folded client; (2) RPAP3 tethers Hsp90 *via* its interaction with the C-terminal MEEVD motif; (3) PIH1D1 engages the client or an adaptor protein. (4) RUVBL1/2 use ATP hydrolysis to remodel the client and/or recruit additional assembly factors.

Together, these interactions create modular and flexible chaperone machinery where folding, stabilization, and complex assembly are integrated into a single coordinated process. During the ATP-driven conformational transitions of Hsp90, mechanical tension may be transmitted through RPAP3 to the PIH1D1-RUVBL1/2 interface, thereby synchronizing client release with the ATP-dependent rearrangements of the RUVBL1/2 ring. PIH1D1 serves a dual purpose by functioning as both a connector and a molecular sensor.

In this architecture, Hsp90 provides folding capacity, RPAP3 acts as a scaffold and signal conduit, PIH1D1 offers selectivity, and RUVBL1/2 drives structural remodeling. Overall, this cooperative network enables the R2TP system to integrate diverse proteostatic pathways, thereby supporting the maturation of large and structurally complex macromolecular assemblies.

### Major client classes managed by the Hsp90-R2TP axis

Table 1 summarizes the main client classes of the Hsp90-R2TP machinery, their biological functions, and the adaptor proteins that mediate their recruitment. Collectively, these client groups highlight the extensive range of functions of the R2TP-Hsp90 system. RPAP3 acts as the principal scaffold that couples the ATP-driven folding cycle of Hsp90 to the remodeling and assembly of the RUVBL1/2 ATPases.

**Table 1:** Major clients of the R2TP-Hsp90 network and their adaptor modules

Client class	Proteins	Key R2TP interaction	Functional mechanism	RPAP3 involvement^a^
RNA Polymerases	RNA Pol II (RPB1-12 subunits), RNA Pol I, RNA Pol III	RPAP3; PIH1D1; RUVBL1/2	Assembly and nuclear import of RNA polymerase complexes; R2TP promotes subunit stabilization and holoenzyme maturation (13, 14).	Direct, as a scaffold for RNA Pol II
snoRNPs	Box C/D (NOP58, NOP56, fibrillarin, SNU13), Box H/ACA (DKC1, NHP2, NOP10, GAR1)	RPAP3; PIH1D1	Assembly of small nucleolar RNPs is essential for rRNA modification (ribosome biogenesis); R2TP acts as a remodeling platform, ensuring correct RNP architecture (15, 31, 75).	Indirect
miRNAs	TRPB (dsRBD3)	RPAP3	miRNA-dependent regulation; RPAP3 likely sequesters TRBP away from Dicer, thus affecting miRNA maturation and/or subsequent activity (76).	Indirect
PIKK Signaling Kinases	mTOR, ATM, ATR, SMG-1, TRRAP, DNA-PKc	RPAP3; PIH1D1; RUVBL1/2	Assembly and stabilization of large kinase complexes; TELO2 recruits PIKKs to Hsp90-R2TP for folding and activation (17, 21). TELO2-TTI1-TTI2 complex with RUVBL1-RUVBL2 inhibits its ATPase activity and antagonizes RPAP3/Tah1p-PIH1D1/Pih1p engagement (77).	Indirect
TSC complex	TSC1, TSC2, TBC1D7	RPAP3; PIH1D1; RUVBL1/2; WDR92	Regulates the Hsp90 ATPase activity, and the TSC complex is stabilized through the interaction with R2TP subunits (78).	Direct: TPR binding, and C-terminal domain (CD)
Spliceosome snRNP Assembly	PRPF8, EFTUD2, Brr2 (SNRNP200)	RUVBL1/2; RPAP3; PIH1D1	Chaperoning of U5 snRNP core components; R2TP regulates spliceosome maturation and recycling (79, 80, 81).	Direct: C-terminal domain (CD)
Axonemal Dynein Arm Assembly	DNAAF2, DYX1C1, DPCD	PIH1D2; SPAG1; RUVBL2	Assembly of axonemal dynein arms and motile cilia; R2SP (SPAG1-PIH1D2) complex mediates specialized HSP90-dependent maturation of client proteins (45).	Direct: C-terminal domain (CD)
Chromatin and Transcriptional Regulators	TIP60 complex, INO80, SRCAP	RPAP3; PIH1D1; RUVBL1/2	Remodeling of chromatin and transcriptional complexes; R2TP maintains their structural integrity and activity (23).	Indirect
ZNHIT complex	ZNHIT2, ZNHIT3, ZNHIT4, ZNHIT6	RPAP3; RUVBL1/2	NUFIP/ZNHIT3 and ZNHIT6 stabilize the RUVBL1/2: NOP58 complex (82). Participates in U5 snRNP assembly, regulates the ATPase activity of the R2TP co-chaperone, and contributes to substrate specificity (81)	Indirect
Prefoldin-like subunit	URI1	RPAP3; RUVBL1/2; WDR92	Cytoskeleton protein complex assembly; regulates signaling pathways and cell growth (83).	Indirect
Other Emerging Clients	Liprin-α2, WDR92, NUFIP, phospho-regulatory scaffolds	PIH1D2; SPAG1; RPAP3	Scaffold assembly for cell adhesion and migration; illustrates R2TP network adaptability to cell-type-specific functions (45).	Both

a RPAP3 involvement is classified as: (i) direct, *via* TPR or C-terminal contacts, or (ii) indirect, *via* intermediary adaptor proteins that bridge the client to R2TP/RPAP3.

## Disease implications

Dysregulation of the R2TP complex, particularly through altered RPAP3 expression or function, has been increasingly implicated in human disease. As RPAP3 serves as a central scaffold that coordinates the assembly of several essential macromolecular complexes, its perturbation can disrupt proteostasis, transcriptional regulation, and signaling fidelity. Such defects create cellular vulnerabilities that contribute to pathogenesis.

Evidence linking RPAP3 to cancer biology and its potential as a therapeutic target has emerged from studies showing that compounds with antitumoral activity directly bind to RPAP3. Two phenolic marine sesquiterpenes, dictyoceratin-A and -C, which induce hypoxia-selective growth inhibition in cultured cancer cells and display antitumor activity *in vivo*, were shown to bind RPAP3 (68). In experiments in which RPAP3 was knocked down, there was a reduction in cell growth under hypoxic conditions and a decrease in the expression of HIF-1α (a key regulator of hypoxic responses), HK2, and VEGF. These results suggest that dictyoceratin compounds inhibit the RPAP3-Hsp90 interaction, thereby compromising the homeostasis of specific client proteins.

Another RPAP3 knockdown study, performed in breast cancer cell lines (69), showed that depletion of RPAP3 markedly increased survival of cells treated with doxorubicin, whereas RPAP3 overexpression enhanced doxorubicin-induced cell death. In line with this, multiple breast cancer cell lines exhibit elevated NF-κB DNA-binding activity, and RPAP3 overexpression suppresses doxorubicin-induced NF-κB activation. However, additional studies are required to elucidate the role of RPAP3 in these cell lines.

Additionally, high RPAP3 expression in intestinal tumors correlates with poor prognosis, whereas RPAP3 maintains intestinal epithelial homeostasis in proliferative stem cells and progenitors (70). Together, these findings suggest that RPAP3 may be a promising therapeutic target and prognostic biomarker for enhancing the efficacy of anticancer treatments in breast and colorectal cancers. A variant of RPAP3, R26L, a highly conserved residue, is linked to normal-pressure glaucoma (NPG) (71, 72), a disorder characterized by progressive degeneration of the optic nerve. This RPAP3 variant was absent in all unaffected individuals and segregated with the disease phenotype in all but two affected family members, indicating a possible cause of NPG. RPAP3 is likely associated with the neurodegenerative component of the disease.

Components of the R2TP complex, including RPAP3 and PIH1D1, also interact with the L proteins of mumps virus (MuV) and measles virus (MeV), potentially promoting viral polymerase activity (73). In this study, RPAP3 knockdown enhanced MeV propagation but decreased MuV propagation, likely by triggering a stronger immune response against MuV. Although these observations suggest different roles for RPAP3 in viral replication, the underlying mechanisms remain to be elucidated.

## Conclusions

Client proteins demonstrate the wide scope of the R2TP-Hsp90 system, spanning nuclear transcription machineries, snoRNPs, cytoplasmic signaling kinases, and large PIKK complexes. By coupling Hsp90’s conformational cycle with RUVBL1/2-mediated remodeling, R2TP provides a highly adaptable platform for the maturation of structurally diverse substrates. This versatility helps explain the deep evolutionary conservation of R2TP as a central integrator of eukaryotic proteostasis.

Recent years have seen significant advances in understanding RPAP3’s functional role within this machinery, supported by structural, biochemical, and cellular studies from multiple groups. Nevertheless, several aspects of R2TP biology remain unresolved. While the structural and evolutionary foundations of the R2TP-Hsp90 system have been clarified through cryo-EM, biochemical assays, and comparative genomics, several areas require further exploration to translate these insights into therapeutic and biological applications. Therapeutically, selective inhibition of R2TP-Hsp90 interfaces (such as small molecules targeting the RPAP3-PIH1D1 junction) holds promise for disrupting oncogenic PIKK assembly without broadly impairing Hsp90's housekeeping functions.

In conclusion, the potential incorporation of multiple RPAP3 molecules into a single complex suggests additional regulatory layers, yet the *in vivo* stoichiometry and its functional consequences are still unclear. Furthermore, the mechanisms governing Hsp90-R2TP regulation in physiological and pathological contexts are only beginning to emerge, and their relevance for disease intervention remains largely unexplored.

Finally, we acknowledge that a manuscript addressing related aspects was published (74) prior to the submission of the present work. As also noted by the reviewers, we consider that study to provide a valuable complement to ours.

## Conflict of interest

The authors declare that they have no conflicts of interest with the contents of this article.
